# CONCOMITANT CUTANEOUS METASTATIC TUBERCULOUS ABSCESSES AND MULTIFOCAL SKELETAL TUBERCULOSIS

**DOI:** 10.4103/0019-5154.43208

**Published:** 2008

**Authors:** Betul Sezgin, Ulviye Atilganoglu, Ozgul Yigit, Selma Sönmez Ergün, Nevin Cambaz, Cuyan Demirkesen

**Affiliations:** *From Department of Pediatry, Cerrahpasa Medical School, Istanbul, Turkey*; 1 *From Department of Dermatology, Cerrahpasa Medical School, Istanbul, Turkey*; 2 *From Department of Plastic and Reconstructive Surgery, Cerrahpasa Medical School, Istanbul, Turkey*; 3 *From Department of Pathology, Cerrahpasa Medical School, Istanbul, Turkey*

**Keywords:** *Cutaneous metastatic tuberculous abscess*, *skeletal tuberculosis*, *treatment*

## Abstract

Tuberculosis, one of the oldest diseases known to affect humans is caused by the bacteria mycobacterium tuberculosis. The disease usually affects the lungs, although, in up to one third of cases, other organs are involved. Metastatic tuberculosis abscess is a rare form of skin tuberculosis. It is characterized by nodule and abscess formation throughout the body after hematogenous spread of mycobacterium tuberculosis from a primary focus during a period of impaired immunity. Tuberculosis osteomyelitis is also a rare form of extrapulmonary tuberculosis in pediatric age group. Skeletal tuberculosis pathogenesis is related to reactivation of hematogenous foci or spread from adjacent paravertebral lymph nodes. Weight-bearing joints are affected most commonly. Bilateral hand and foot bone involvement is rarely reported. We present a five-year-old girl with two very rare presentations of the disease such as osteomyelitis and metastatic skin abscess.

## Introduction

Although tuberculosis is an ancient disease that is known to have existed in prehistoric times, it remains one of the most important infectious diseases today in terms of morbidity, mortality, and economic impact. The development of resistance to antituberculosis drugs and conditions associated with immunodeficiency such as AIDS and chemotherapy are responsible for the recent increase. As a result unusual presentations of tuberculosis have been encountered more frequently such as cutaneous tuberculosis, skeletal tuberculosis, tuberculous meningitis, tuberculous lymphadenitis and abdominal tuberculosis. In the last decade, a 50% increase in admissions for extrapulmonary tuberculosis was reported.^1^–^7^

Skin tuberculosis, a form of the extrapulmonary tuberculosis, occurs in 1% of all tuberculosis cases. Metastatic tuberculosis abscess is a rare form of cutaneous tuberculosis resulting in single or multiple cutaneous and subcutaneous lesions on trunk, extremities, or head.^3^,^8^,^9^

Skeletal involvement occurs in approximately 1-3% of patients with tuberculosis. The most frequently involved site is vertebrae followed by pelvis and knee joints. Few cases are reported in the literature that showed involvement of tarsal and carpal bones.^3^,^8^,^10^–^12^

We present a five-year-old girl with both metastatic subcutaneous skin abscesses in multiple sites and tuberculosis osteomyelitis of tarsal and carpal bones treated successfully by antituberculosis agents.

## Case History

**Fig. 1:**
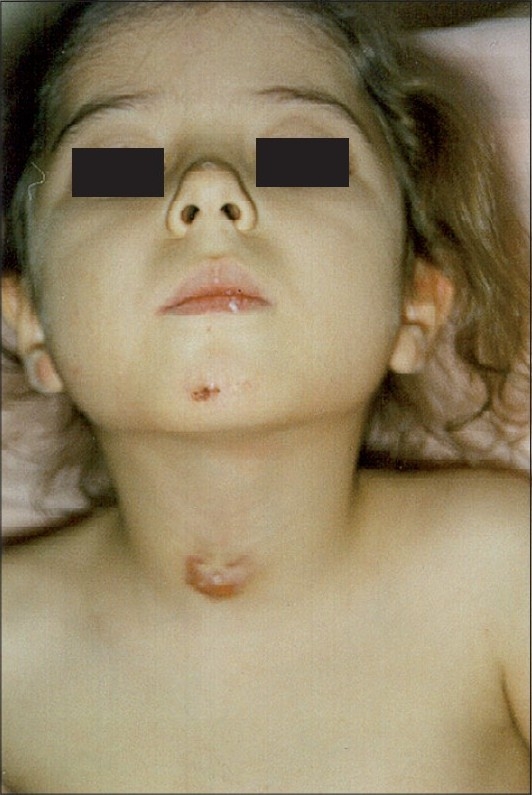
Cutaneous metastatic skin abscess on the neck

**Fig. 2:**
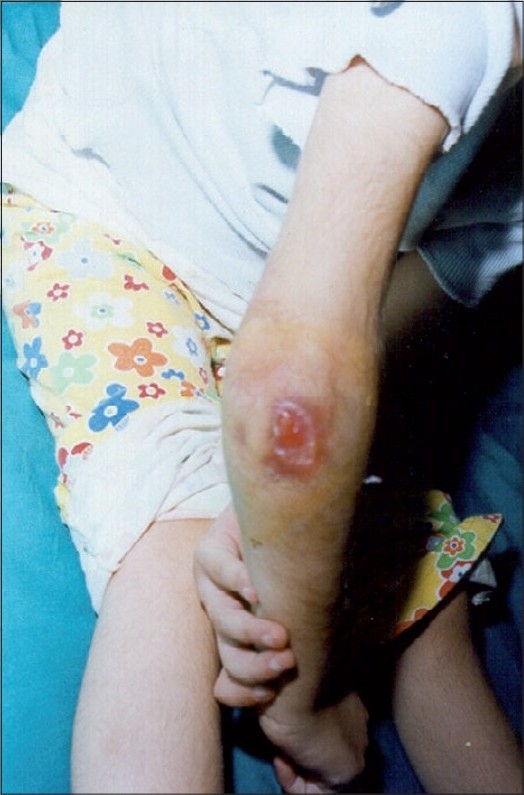
Cutaneous metastatic skin abscess on right elbow

**Fig. 3:**
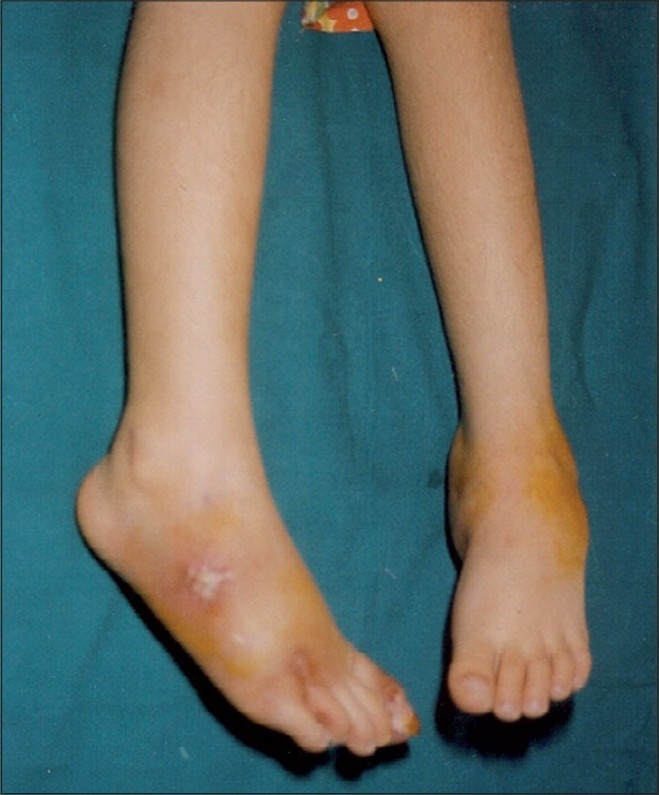
Bilateral cutaneous metastatic skin abscess on dorsum of the foot

A five-year-old malnourished female patient was admitted to our hospital with multiple subcutaneous abscesses on her neck, right elbow, left fourth finger, left lateral malleolus, left and right dorsum of the foot and right great toe, which had been present for seven months (Figs. 1–3). After she received courses of antibiotics, the lesions, initially pea-sized, failed to heal; on the contrary, they enlarged.

Dermatologic examination revealed widespread dusky red nodules. The lesions were fluctuating and non-tender without redness or local warmth on the overlying skin.

The left fourth finger, right elbow, left ankle and bilateral dorsal foot regions were swollen and painful. Regional lymphadenopathy was not detected. The other physical findings were normal.

Her weight and height were 15 kg and 100 cm respectively (both below the third percentile). There was history of progressive weight loss. She had no previous history of skin injury or immunosuppressive therapy. The family history revealed a past occurrence of treated pulmonary tuberculosis in one of the house members. She did not have BCG vaccine. A quantative reading of PPD (purified protein derivate) skin test was positive with 22 × 29 mm of induration at the 72^nd^hour. Erythrocyte sedimentation rate was 102 mm/hr. Blood count showed signs of pancytopenia (HGB: 9.2 gr/dl, WBC: 3260/mm³, PLT: 100 000/mm³). CRP was 4.05 mg/dl (usual value 0-1 mg/dl). Serology for antibodies to HIV was negative.

**Fig. 4:**
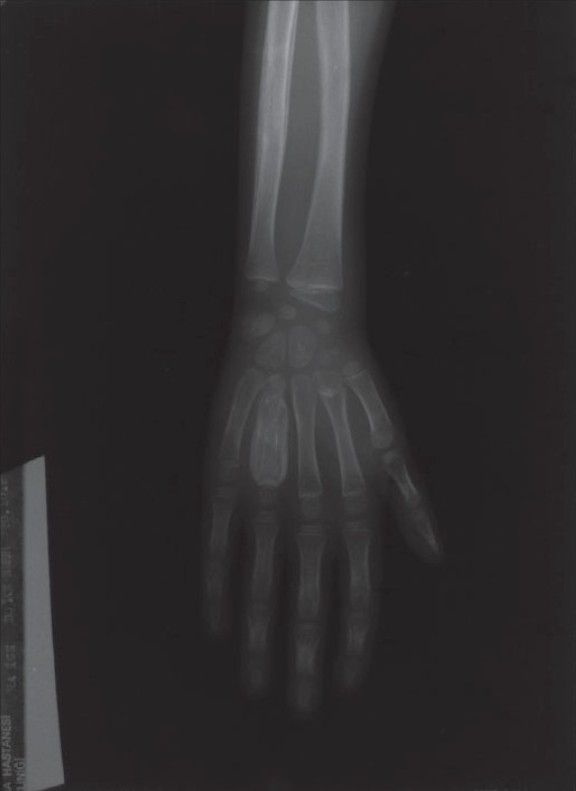
Fusiform, expansive heterogenous material filling the diaphysis of the fourth metacarpal bone describing spina ventosa

**Fig. 5:**
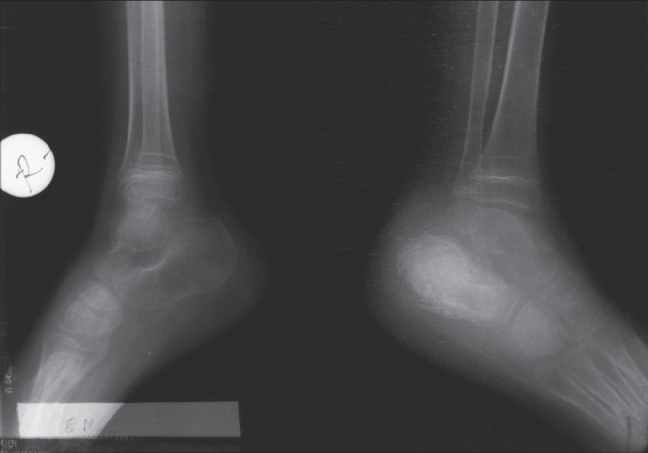
“Bone in bone” pattern describing calcaneal bony expansion with sclerotic changes in the center surrounded by a radiolucent ring representing secondary changes due to medullary involvement of tuberculosis

**Fig. 6:**
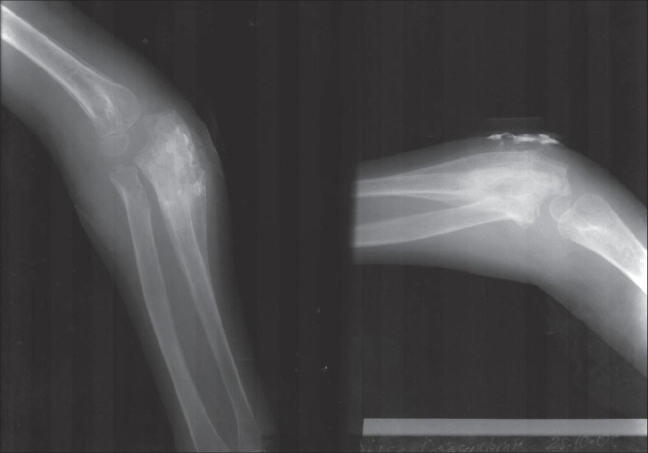
Increased medullary intensity resulting from subperiostal expansion in the area between metaphysis and diaphysis in ulna consistent with tuberculosis osteomiyelitis

The chest roentgenogram was normal. The roentgenograms of the extremities showed a periosteal reaction in the left fourth metacarpal bone, right proximal ulna, right proximal humerus, left distal fibula and tibia and bilateral calcaneus (Figs. 4–6). The findings on the bone scan were suggestive of osteomyelitis.

**Fig. 7:**
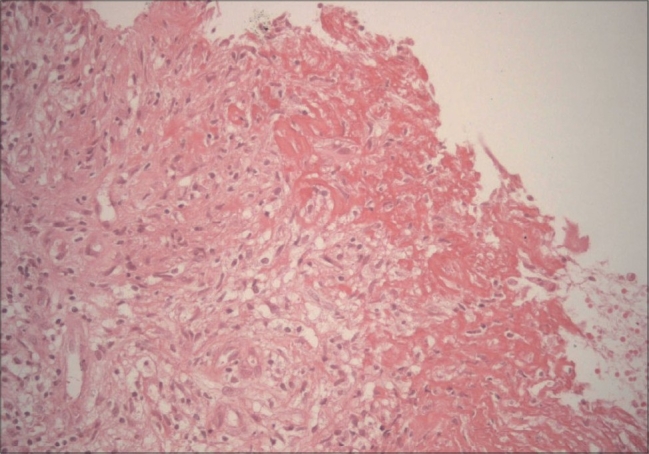
Skin biopsy revealing large areas of necrosis, surrounded by palisading epitheloid histiocytes (H&E, ×100)

Skin biopsy specimen was taken from the margin of the active skin lesion. Large areas of necrosis, surrounded by palisading epithelioid histiocytes, Langhans-type multinucleated giant cells and lymphocytes were detected in this skin biopsy (Fig. 7). Using Ehrlich-Ziehl-Neelsen stain, acid-fast bacilli were not seen.

On the basis of clinical-pathological findings, positive tuberculine skin test, and history of close contact with tuberculosis patient in her family, a final diagnosis of extrapulmonary tuberculosis was made.

Abscesses were drained and treatment with isoniazid (INH) 15 mg/kg/day, rifampicin (RMP) 15 mg/kg/day, ethambutol (EMB) 15 mg/kg/day and pyrasinamid (PZA) 20 mg/kg/day were started. The lesions regressed within two months. After two months EMB and PZA were discontinued. INH and RMP were given for 16 more months.

Three months later, her symptoms waned, and the abnormal results on laboratory tests and findings on extremity roentgenograms markedly improved. Complete resolution occurred by nine months. At the end of 60 months follow-up, the patient was clinically very healthy.

## Discussion

Tuberculosis has been known to affect mankind since the dawn of human civilization and still remains a major problem in the developing countries. Extrapulmonary tuberculosis accounts for up to one third of all cases. Children show a higher predisposition to the development of extrapulmonary tuberculosis.^3^,^8^,^9^

The overall incidence of cutaneous tuberculosis among all forms of tuberculosis is approximately 1-2%. M. tuberculosis, m.bovis and under certain conditions the attenuated BCG organism cause all forms of cutaneous tuberculosis. Classification has been attempted according to morphology, and more recently the mode of infection or the immunologic state of the host. Although primary inoculation tuberculosis and tuberculosis verrucosa cutis are exogenous infections; lupus vulgaris, scrofuloderma, metastatic tuberculous abscess, acute miliary tuberculosis and orificial tuberculosis are endogenously spread cutaneous tuberculosis forms. Kumar *et al*, reported that lupus vulgaris was the most common clinical presentation, followed by scrofuloderma, tuberculosis verrucosa cutis and cutaneous metastatic tuberculous abscess (tuberculous gumma) in their series.^1^,^3^,^6^,^8^,^13^

Metastatic tuberculous abscesses may occur along with progressive organ tuberculosis or in miliary tuberculosis, however there are reports showing that it may occur without any underlying tuberculous focus as occurred in our patient. It usually occurs in undernourished children of low socioeconomic status or immunodeficient or severely immunosuppressed patients. Our patient had a lower socioeconomic status, too, her general condition was consistent with failure to thrive and she had manifestations of malnutrition.^3^,^8^,^9^,^14^

The differential diagnosis includes staphylococcal abscess, other mixed bacterial infections, spirotrichosis, nocardiosis, chromomycosis, leishmaniasis, atypical mycobacterial infections, deep fungi infections, syphilitic gumma, leprosy and all forms of panniculitis. Confirmation of the clinical diagnosis is obtained by histopathology, and bacterial or fungal culture.^8^,^15^,^16^

Tuberculin sensitivity is usually lower than in other forms of skin tuberculosis and may be absent in severely ill patients.^1^,^5^,^6^,^8^,^13^,^14^ There was strong tuberculine positivity in our patient that could be attributed to concomitant tuberculosis osteomyelitis.

Skeletal tuberculosis accounts for 10-15% of cases of extrapulmonary tuberculosis.^15^,^17^,^18^ Recently, the proportion of bone or joint tuberculosis has progressively increased to 27%.^19^ Although they are highly specific in the presented case, the clinical findings may not be specific, and in many cases, the radiographic appearance is similar to pyogenic osteomyelitis; therefore, the disease is frequently neglected or misdiagnosed. The symptoms are those usually seen in musculoskeletal disorders; pain, stiffness, and swelling. The tuberculin test is positive in up to 90% of cases.^2^,^7^,^12^,^15^,^20^

Multifocal bone involvement can occur but it is occasionally reported in children. The spine and hip are the most common sites of osteoarticular tuberculosis. On the other hand, tuberculosis of the hand and foot is less common. The spine is involved in half the cases, whereas dactylitis contributes only to 4% of cases.^2^,^7^,^12^,^18^,^19^,^21^

Tuberculous involvement of the short, tubular bones of the hands and feet is termed tuberculous dactylitis. Fusiform soft-tissue swelling and periostitis are the most common radiographic findings. As underlying bone is destroyed, a cyst-like cavity forms and the remaining bone appears to be ballooned out. This appearance is termed spina ventosa. In children, tuberculous dactylitis can produce spina ventosa like our patient, a pattern of chronic osteitis characterized by central defect, soft tissue swelling, and exuberant periosteal reaction.

The most common radiologic finding is that of osteoporosis, which may be intense cancellous bone involvement and may present as a cystic lesion with or without sequestrum.^7^,^10^,^12^,^15^

Roentgenograms, CT or MRI reveal the characteristic lesion and suggest its etiology, although the differential diagnosis includes other infections and tumors. Aspiration of the abscess or bone biopsy confirms the tuberculous etiology, as histologic findings are highly typical and cultures are usually positive; however skeletal and skin tuberculoses are paucibacillary lesions and it is difficult to demonstrate or culture acid-fast mycobacteria from these lesions like our patient.^22^

The incidence of active pulmonary tuberculosis associated with skin or skeletal tuberculosis is reported as 50-65% in the literature. The concomitant presentations of skin and skeletal tuberculosis is a very rare condition. Kivanc-Altunay *et al*, reported that 61% of skin tuberculosis cases had pulmonary involvement whereas no skeletal tuberculosis was encountered. Kumar *et al*, reported that four patients out of 75 with skin tuberculosis had skeletal tuberculosis and only one had tuberculous dactylitis. Although active pulmonary tuberculosis was not present in our case, two rare presentations of tuberculosis, skin tuberculosis and skeletal tuberculosis were concomitantly observed.^4^,^6^

The histological findings of metastatic tuberculous abscess involve massive necrosis and abscess formation with or without acid fast bacilli. No bacilli were shown among typical necrotising granulomas in our case. The culture and PCR results were also negative for tuberculosis in our case. In endemic regions, the clinical features, radiological appearance and elevated ESR are sufficient to diagnose tuberculosis and begin treatment.^10^,^15^,^23^,^24^ Considering positive family history with a patient having cavernous tuberculosis in the same home environment, strong positive PPD results, X-ray findings and necrotising granulomas in histology, metastatic tuberculous abscesses and tuberculous osteomyelitis were diagnosed in our case. Immune suppression due to malnutrition was the explanation for the clinical situation.

In conclusion, tuberculosis incidence has increased in industrialized countries, which is attributed to several factors such as immigration from countries with a high prevalence of tuberculosis, infection with HIV, emergence of multi-drug resistant tuberculosis, and social problems such as poverty, homelessness and drug abuse.^22^ Rare extrapulmonary tuberculosis cases are seen more frequently as a result of this. The possibility of tuberculosis must always be kept in mind in subcutaneous abscess and nodule cases non-responsive to non-spesific therapy, and drainage. Awareness of the unusual presentations of tuberculosis is essential for early diagnosis and proper therapy. Therefore physicians must have a high index of suspicion with regard to tuberculosis.
